# First detection of tick-borne encephalitis virus in *Ixodes ricinus* ticks in Belgium, May 2024

**DOI:** 10.1186/s13071-025-06829-5

**Published:** 2025-05-30

**Authors:** Camille Philippe, Celine De Sterck, Anna Parys, Sarah Denayer, Nick De Regge, Gabrielle Trozzi, Tinne Lernout, Marcella Mori, Bert Devriendt, Eric Cox, Sanne Terryn, Steven Van Gucht, Hein Sprong, François E. Dufrasne

**Affiliations:** 1 Sciensano, Belgian Health Institute, Brussels, Belgium; 2 Present Address: Laboratory of Immunology, Department of Translational Physiology, Infectiology and Public Health, Faculty of Veterinary Medicine, Ghent University, Merelbeke, Belgium; 3 Centre for Infectious Disease Control, National Institute for Public Health and Environment (RIVM), Bilthoven, The Netherlands

**Keywords:** Tick-borne encephalitis, Tick-borne encephalitis virus, Ticks, Vector-borne diseases, *Ixodes Ricinus*

## Abstract

Tick-borne encephalitis (TBE) is the most frequent tick-borne viral disease transmitted by ticks in Europe and Asia. In Belgium, autochthonous cases of TBE have been reported, but even though some tick collection was carried out in the past, no TBEV-positive ticks have been found thus far. In this study, questing ticks were collected by flagging at the precise location where a patient was reported to have been bitten by a tick before developing TBE in Belgium in 2020. In total, 350 ticks were pooled by life stage (nymphs, adult females, adult males) and collection date, lysed, and RNA extracted. Quantitative reverse transcription polymerase chain reaction (RT-qPCR) was performed to detect tick-borne encephalitis virus (TBEV) and *Ixodes* 18S rRNA, followed by Oxford nanopore amplicon sequencing. TBEV was detected in all three types of pools. Out of 69 nymph pools, 2 were positive, in adult female pools, 2 out of 16 were positive, and 1 of the 14 adult male pools was positive. A complete sequence was retrieved through sequencing. This sequence shares greater similarity with a strain found in Finland than the neighboring Salland strain (the Netherlands) and the Neudoerfl reference strain. These findings confirm that TBE can be acquired from tick bites within the country. It is therefore necessary to increase awareness of the disease among healthcare professionals.

**Graphical abstract:**

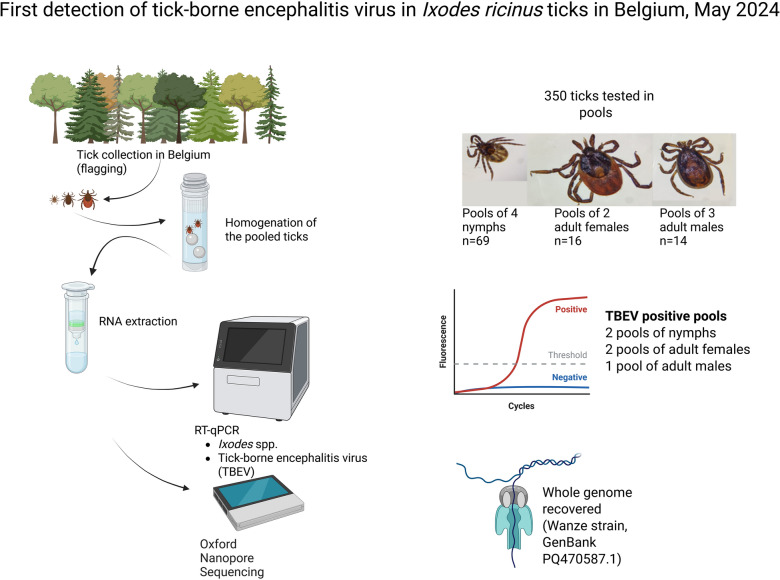

**Supplementary Information:**

The online version contains supplementary material available at 10.1186/s13071-025-06829-5.

Tick-borne encephalitis virus (TBEV) is a flavivirus that can cause encephalitis in humans and some domesticated animals. It is the most frequent tick-borne viral disease reported in Europe and Asia. Ticks act as vectors and reservoirs for TBEV, while small mammals serve as reservoirs and amplifying hosts, thereby increasing the number of TBEV-infected ticks. Humans are considered accidental or dead-end hosts, typically infected through a tick bite, or more rarely, by consuming unpasteurized dairy products [1].

It is estimated that two-thirds of TBEV infections in humans are asymptomatic. In symptomatic cases, the first phase of the disease is characterized by flu-like symptoms. In 20–30% of those infected, a second phase develops, potentially leading to meningitis, meningoencephalitis, and other central nervous system complications, which can lead to permanent sequelae [1].

Tick-borne encephalitis (TBE) is increasingly recognized as a public health challenge in Europe and other parts of the world. Between 1973 and 2003, the number of human cases of TBE in all endemic regions of Europe increased by nearly 400% [2]. While notification rates have remained relatively stable over the past 20 years, with occasional peak years, the areas at risk have expanded, and new foci of the disease have been discovered. Indeed, TBEV was only relatively recently detected in the United Kingdom and the Netherlands [4, 5].

Different factors predict the risk of TBEV infections, including human behavior, climatic factors such as the average winter temperature and total annual precipitation, and environmental factors such as wildlife reservoirs that can influence tick populations (e.g., rodents and cervids) [6].

In Belgium, serological studies in several animal species suggest that TBEV has been circulating for more than a decade [7–9]. In 2020, three autochthonous human cases of TBE were detected [10]. Tick collection has been carried out in Belgium for several years, and at least 7000 ticks have been tested for TBEV. These ticks were either questing, collected by flagging (*n* = 4884), or removed from humans (*n* = 2153). However, no TBEV-positive ticks were identified [7, 11–13]. Therefore, until now, only indirect evidence has been available for the circulation of the virus.

To investigate the circulation of TBEV in Belgium at possible hotspots, ticks were collected at the precise location where one of the three cases was bitten in 2020 [10]. Questing ticks were collected by flagging in May 2024 at the Bois de Champia (Wanze, Belgium, 50°33′50.7"N 5°14′50.8" E). A total of 350 *Ixodes ricinus* ticks were collected and tested. Ticks were pooled by collection date and life stage: nymphs were grouped by four, adult females in pairs, and adult males by three, and placed in a tube containing Dulbecco Phosphate-buffered saline (DPBS) and two metallic beads with a diameter of 5 mm. Ticks were lysed using a tissue lyser at 25 Hz for 5 min. Part of the homogenised material was used for RNA extraction, while another was used to infect Vero and BHK-21 cell lines. A multiplex RT-qPCR targeting TBEV and *Ixodes* 18S rRNA was conducted [14].

TBEV was detected in all three types of pools; 2 of 69 nymph pools were positive (2.90%, 95% CI 0.35–10.80%). In the adult female pools, 2 out of 16 were positive (12.50%, 95% CI 1.55–38.35%), and 1 of the 14 adult male pools was positive (7.14%, 95% CI 0.18–33.87%). Two highly positive pools were sequenced by amplicon sequencing using Oxford Nanopore Technology [15]. Ct values for positive and sequenced pools are provided in Additional file 1: Table S1. A complete sequence was retrieved and designated “Wanze” as TBEV was detected in that municipality in Belgium. The accession number for this strain is PQ470587. Phylogenetic comparison with other European strains showed that the TBEV Wanze strain is closely related to a strain found in Finland (Sipoo archipelago) and is distinct from the Salland strain (identified in the Netherlands) and the Neudoerfl reference strain (Fig. 1) [16, 17]. The Wanze and Salland strains differ by 73 amino acids, while Wanze and Neudoerfl differ by 26. The smallest difference is observed between Wanze and Sipoo, with only 11 amino acid variations (Fig. 2).

**Fig. 1:**
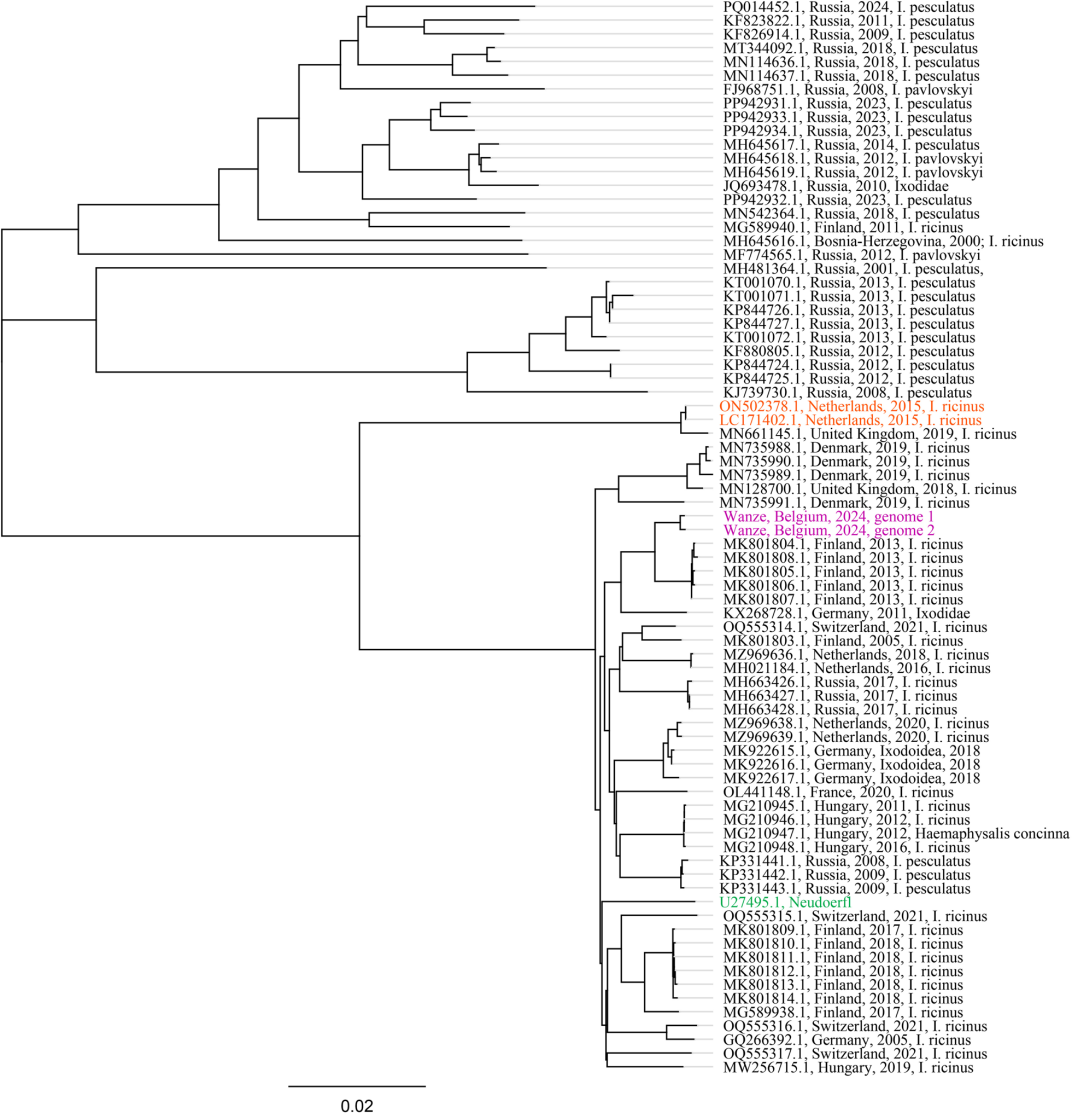
Phylogenetic tree of all European TBEV strains found in ticks from 31 December 1999 until 1 September 2024 on NCBI Virus, TBEV-Wanze, and Neudoerfl. Orange sequences are the Salland strains. Purple sequences are the newly sequenced TBEV Wanze Belgian strains. Green sequence is the Neudoerfl reference strain

**Fig. 2:**
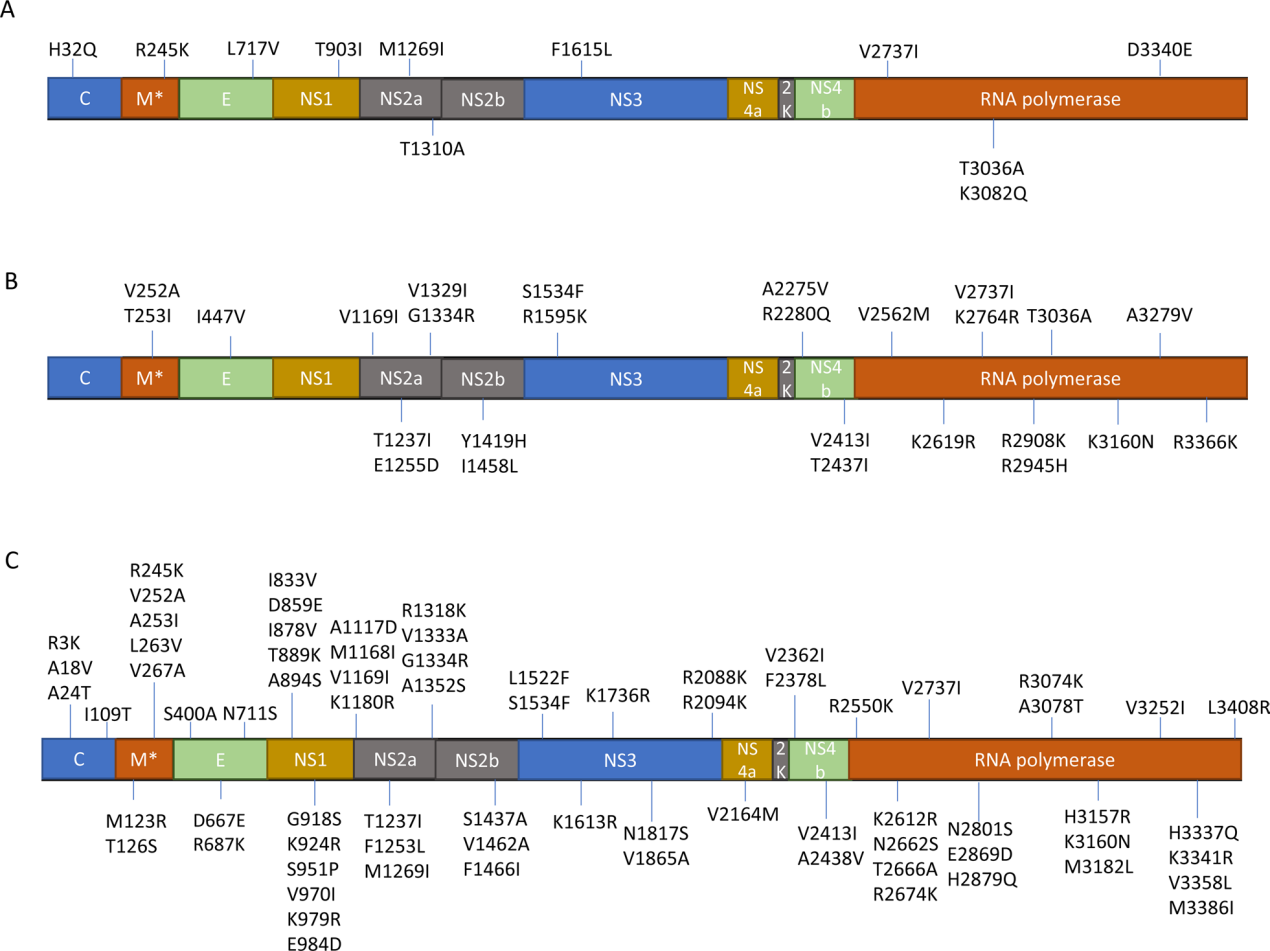
Comparison of amino acid differences in the polyprotein sequences of TBEV Wanze and other TBEV strains. **A** Sipoo strain, **B** Neudorfl strain, **C** Salland strain

The Salland strain is closely related to a strain found in the United Kingdom, possibly due to migratory birds’ carriage of infected ticks. This hypothesis could also explain the similarity between the Belgian strain (Wanze) and the one found in Finland [4, 17].

These findings mark the first identification of an endemic TBEV focus in Belgium and confirm that the disease can be acquired from tick bites within the country. Thus, they contribute to a broader understanding of the geographic spread of TBEV in Europe.

The detection of TBEV at the location where a patient was bitten 4 years ago suggests long-term circulation of the virus in this area. Additionally, the presence of TBEV across multiple tick life stages further strengthens the evidence that the virus has been circulating in that area for several generations of ticks.

The main limitations of this study are the relatively small sample size, with only 350 ticks collected and tested from a single year and a single forest, and as in other studies, the use of pooled tick samples. While pooling ticks allows for more efficient and cost-effective testing, it may lead to underestimating TBEV prevalence in ticks, as multiple ticks are grouped into a single sample. This approach can also result in viral dilution, making detection more difficult in pools with low viral concentrations. To more accurately estimate the true prevalence of TBEV, future studies should focus on testing a larger number of individual ticks, collected over multiple years and from different forests.

Healthcare professionals and clinical microbiologists should be aware that TBEV circulates in Belgium and should consider TBE(V) in their differential diagnosis. Currently, vaccination for TBEV in Belgium is only recommended for travelers to other countries with outdoor activities in high-risk areas [18]. The vaccination strategy is based on different scenarios, considering the epidemiological situation of endemic TBE cases. In the context of sporadic cases of TBE, which are geographically spread, vaccination is currently not recommended for the general population, nor are professional or recreational groups at risk. However, human cases are likely underdiagnosed, as mild infections often go undetected, and laboratory investigations for viral encephalitis are not systematically performed. To better document the epidemiological situation of TBE in Belgium, it is thus essential to conduct further research on TBEV in ticks and have a sustained nationwide monitoring system of the circulation of the virus in animals. It is also necessary to increase awareness of the disease among healthcare professionals, especially in patients with a recent history of tick bite and/or encephalitis of unknown etiology.

## Supplementary Information

Additional file 1

## Data Availability

The two Wanze TBEV sequences were submitted to GenBank. The accession numbers for these sequences are PQ470587 and PQ492320.
