# Supplementary material for: First detection of tick-borne encephalitis virus in Ixodes ricinus ticks in Belgium, May 2024
Source: Parasit Vectors. 2025 May 30;18:197. doi: 10.1186/s13071-025-06829-5 (PMC12125855; doi:10.1186/s13071-025-06829-5)
Supplement: Supplementary file 1 — Additional file 1 [file 13071_2025_6829_MOESM1_ESM.docx]

| **Positive pool** | **Pool composition** | **ct value** | **Sequenced** |
| --- | --- | --- | --- |
| 1 | 4 nymphs | 29,91 |  |
| 2 | 4 nymphs | 31,67 |  |
| 3 | 2 adult females | 16,03 | Yes |
| 4 | 2 adult females | 17,42 | Yes |
| 5 | 3 adult males | 33,4 |  |

Table S1. Ct values of positive tick pools and sequenced pools
